# Encephalitis Caused by Pathogens Transmitted through Organ Transplants, United States, 2002–2013

**DOI:** 10.3201/eid2009.131332

**Published:** 2014-09

**Authors:** Sridhar V. Basavaraju, Matthew J. Kuehnert, Sherif R. Zaki, James J. Sejvar

**Affiliations:** Centers for Disease Control and Prevention, Atlanta, Georgia, USA

**Keywords:** encephalitis, transplant, transplantation, transmission, West Nile virus, rabies, lymphocytic choriomeningitis virus, Balamuthia mandrillaris, viruses, donor-derived infection, transplant-transmitted pathogen

## Abstract

The cause of encephalitis among solid organ transplant recipients may be multifactorial; the disease can result from infectious or noninfectious etiologies. During 2002–2013, the US Centers for Disease Control and Prevention investigated several encephalitis clusters among transplant recipients. Cases were caused by infections from transplant-transmitted pathogens: West Nile virus, rabies virus, lymphocytic choriomeningitis virus, and *Balamuthia mandrillaris* amebae. In many of the clusters, identification of the cause was complicated by delayed diagnosis due to the rarity of the disease, geographic distance separating transplant recipients, and lack of prompt recognition and reporting systems. Establishment of surveillance systems to detect illness among organ recipients, including communication among transplant center physicians, organ procurement organizations, and public health authorities, may enable the rapid discovery and investigation of infectious encephalitis clusters. These transplant-transmitted pathogen clusters highlight the need for greater awareness among clinicians, pathologists, and public health workers, of emerging infectious agents causing encephalitis among organ recipients.

## Abstract 2

Donor-derived infectious encephalitis among transplant recipients is rare and may not be recognized by clinicians.

More than 500,000 solid organ transplants have been performed worldwide, and >28,000 are performed annually in the United States (*1*). Improvements in immune-modulating therapy, critical care medicine, and surgical techniques have led to the increased success of organ transplantations, and more patients are now eligible for these procedures. In the United States, >100,000 patients are currently on organ transplant waiting lists (*1*).

The risk for infections caused by pathogens transmitted through solid organ or tissue transplants (hereafter referred to as donor-derived or transplant-transmitted infections) has been recognized for decades and remains a worldwide public health problem (*2*). The gravity of these infections took on greater focus after the HIV epidemic emerged in the 1980s (*3*). Infections caused by other donor-derived pathogens in transplant recipients are often asymptomatic or may result in nonspecific signs and symptoms, including unexplained fever or end-organ injury (*4*). Because of immunosuppression and underlying co-existing conditions in transplant recipients, these infections can be severe and fatal. The recognition of this risk led to the screening of donors for some infectious agents (e.g., HIV and hepatitis B and C), which made the organ supply substantially safer. However, a residual transmission risk persists, which might be further reduced by the use of new technologies, such as nucleic acid testing (NAT) (*5*).

Since 2002, several types of emerging donor-derived infections have been reported with increasing frequency among solid organ transplant recipients seeking medical care for encephalitis. These cases can present a diagnostic challenge for clinicians and highlight the need to increase awareness among transplant clinicians regarding the necessity for prompt recognition and treatment of transplant-transmitted infections.

The signs and symptoms of encephalitis vary, depending on the region of the brain involved, but most cases are characterized by global cerebral dysfunction or focal neurologic deficits (*6*). Diagnosing the cause of encephalitis in transplant recipients may be particularly difficult because the cardinal sign of encephalitis (alteration of mental status) can be attributed to numerous other systemic processes. In addition, there are other noninfectious causes of encephalitis, including toxic, metabolic, neoplastic, and autoimmune processes. The signs and symptoms of donor-derived infections can be obscured by co-existing conditions in the transplant recipient, or they can appear more abruptly than in natural infection because of a higher inoculum of organisms and immunosuppression in the transplant recipient. Thus, transplant-transmitted pathogens may be an underrecognized cause of encephalitis.

Since 2002, the US Centers for Disease Control and Prevention (CDC) has investigated clusters of encephalitis among transplant recipients. Cases have been caused by emerging pathogens, including West Nile virus (WNV) (*7*,*8*), rabies virus (*9*), lymphocytic choriomeningitis virus (LCMV) (*10*), and *Balamuthia mandrillaris* amebae (*11*). The cases highlight the difficulties in diagnosing or recognizing clusters of infectious encephalitis among transplant recipients. We review the emerging infectious agents known to cause transplant-transmitted encephalitis, as described from several recent outbreak clusters reported to and investigated by CDC, and suggest methods for better identifying possible donor-derived infections.

## West Nile Virus

WNV, an enveloped, positive, single-stranded RNA flavivirus within the Japanese encephalitis serologic complex, was historically associated with infrequent epidemics of relatively mild febrile illness in parts of Africa, Asia, and Europe (*12*). In 1999, WNV virus was first identified in North America, where it caused an outbreak of encephalitic illness in New York City. Within 5 years, WNV caused the largest epidemic of arboviral encephalitis and became the most common etiologic agent of arboviral encephalitis in the Western Hemisphere. By the end of 2011, >30,000 human cases of WNV encephalitis throughout the United States had been reported to CDC, including >13,000 cases of neuroinvasive disease (*13*).

**Figure 1:**
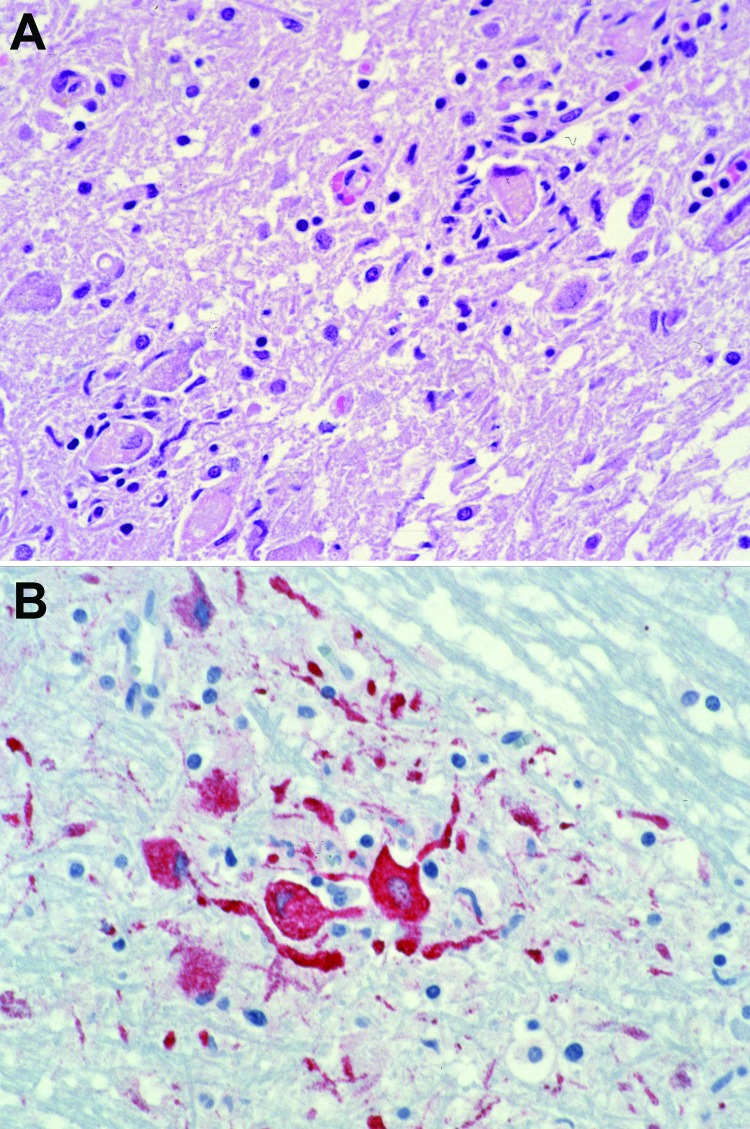
Photomicrographs showing histopathologic features and immunolocalization of West Nile virus antigens in central nervous system tissue from a kidney transplant recipient with transplant-transmitted West Nile virus infection. A) Central nervous system showing mononuclear inflammation, gliosis, and neuronophagia. Hematoxylin and eosin staining. Original magnification, ×125. B) West Nile virus antigens within neurons and neuronal processes. Immunoalkaline phosphate staining, naphthol fast red substrate with light hematoxylin counterstain. Original magnification, ×125.

The natural mode for WNV transmission is through the bite of infected mosquitoes, primarily *Culex* spp. mosquitoes; birds serve as amplifying hosts (*12*). In 2002, several cases of serologically confirmed WNV infection occurred in persons with little or no known exposure to mosquitoes, and epidemiologic evidence suggested transmission of the virus through blood transfusions. Later that year, the first recognized US cases of organ transplant–transmitted WNV were described (*8*). For these infections, the initial link to the transplanted organ was made by histopathologic evaluation and immunohistochemical testing of tissue from an organ recipient who died 4 weeks after undergoing transplantation (Figure 1). However, a link between the infections and the 2 mechanisms of pathogen transmission (i.e., blood transfusion and solid organ transplant) had been suspected early in the epidemic, when WNV infection was diagnosed in 1 of the organ donors who had received a transfusion of WNV-infected blood. In addition, concern was raised over the possibility of a transplant-transmitted infectious agent when illness occurred in 4 recipients of organs from a common donor: febrile illness developed in the liver recipient, and WNV encephalitis developed in 2 kidney recipients and the heart recipient (*8*). Laboratory and epidemiologic data substantiated this mode of virus transmission and documented that the organ donor had likely acquired WNV through a blood transfusion. Subsequent investigation by CDC verified 23 cases in 2002 of WNV acquired through blood or blood components (*7*).

Since blood screening was put into place through pooled donation NAT, there have been 12 published cases of transfusion-transmitted WNV. Of these 12 cases, 5 occurred after blood collection agencies voluntarily implemented processes to trigger more sensitive individual NAT based on local WNV activity (*14*–*16*). There have been no recognized cases of transfusion-transmitted WNV when individual NAT has been used. In 4 of the 5 cases that occurred after testing of individual blood samples was begun, individual testing was not triggered by local events, resulting in breakthrough infection that was not detected by pooled NAT. In another case, individual screening was triggered, and NAT was done, but before the screening results were received, a granulocyte unit was released for transfusion use; testing results later showed that the WNV load was sufficient for detection by NAT of pooled and individual blood samples (*16*).

The 2002 transplant-transmitted WNV cluster heightened awareness of the potential increased severity of WNV infection, particularly neuroinvasive disease, in transplant recipients and immunocompromised persons. It is believed that WNV transmission through blood transfusion has become rare since NAT of donated blood was implemented; however, this may not be the case in the solid organ–transplant setting because organ donor screening has not been mandated.

Six clusters of organ transplant–transmitted WNV were reported to CDC during 2002–2013. In those clusters, WNV infection developed in 12 of 16 transplant recipients; encephalitis developed in 9 of the 12 infected persons, and 4 of those 9 patients died (*17*). It is likely that signs and symptoms of encephalitis among transplant recipients during a WNV outbreak led to the recognition that WNV had been transmitted through organ transplants (*17*). In 2005, another cluster of donor-derived infection was observed among 3 of 4 recipients of organs from a common donor who had naturally acquired WNV infection (*18*).

In the blood transfusion setting, experience has suggested that it is unusual for WNV to be transmitted though the blood of a WNV IgM–positive donor. It has been assumed that a WNV-specific neutralizing antibody response results in clearing of viremia, rendering the blood no longer infectious and probably safe for transfusion use (*19*). However, this may not be the case in the solid organ–transplant setting. The organ donor in the 2005 transplant-transmitted WNV cluster had detectable WNV-specific IgM and IgG but no detectable WNV RNA, suggesting that WNV may be transmitted through transplanted organs from infected persons who have already mounted an antibody response. It may be that transmission is possible because of viral persistence in donated organs after peripheral viremia has cleared or because of intermittent viremia from a reservoir organ, such as a kidney (*20*). Thus, it may prove challenging to implement WNV screening of potential organ donors.

## Rabies Virus

Rabies virus (family *Rhabdoviridae*, genus *Lyssavirus*) is an enveloped, negative, single-stranded RNA virus (*21*). After the virus is inoculated into humans, typically through the saliva or bites or scratches from infected animals, it is taken up through peripheral nerves. Then, through retrograde transport, the virus infects the central nervous system and causes encephalitis; this process may take weeks or months (*21*). Clinical manifestations of rabies virus infection include a nonspecific prodrome (malaise, fever, headache) that lasts days to weeks, followed by confusion and paresthesias, which progress to paresis, hydrophobia, coma, and death (*22*). Rabies is nearly always fatal once neurologic signs develop. In industrialized countries, the disease occurs infrequently because of good veterinary public health practices and postexposure prophylaxis of person exposed or potentially exposed to rabies virus. However, in developing countries, rabies is a common cause of encephalitis (*22*).

**Figure 2:**
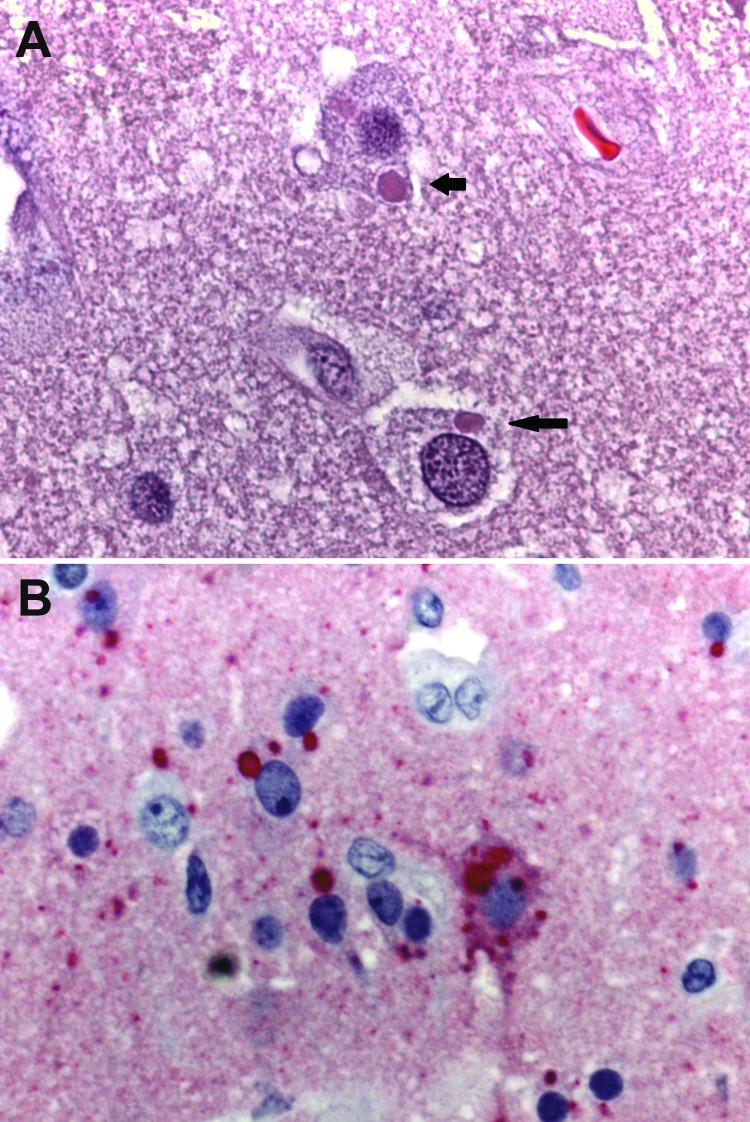
Photomicrographs showing histopathologic features and immunolocalization of rabies virus antigens in central nervous system tissue from a kidney transplant recipient with donor-derived rabies infection. A. Typical intracytoplasmic eosinophilic inclusions (Negri bodies, arrows). Hematoxylin and eosin staining. Original magnification, ×158. B) Rabies virus antigens within neurons and neuronal processes. Immunoalkaline phosphate staining, naphthol fast red substrate with light hematoxylin counterstain. Original magnification, ×158.

The transmission of rabies virus through cornea transplantation has been described, but transmission through solid organ transplantation was not recognized before 2004 (*23*). In July 2004, CDC was notified that 3 recipients of solid organs and 1 recipient of an iliac artery segment from a common donor had died from encephalitis, which was eventually found to be caused by rabies virus infection (*9*). The initial link to rabies virus was made by histopathologic evaluation and immunohistochemical testing of central nervous system tissue (Figure 2). The common-source outbreak of donor-derived rabies infection highlighted the difficulties inherent in the evaluation of encephalopathy in a potential organ donor. Before dying, the donor associated with this outbreak cluster had been evaluated twice in an emergency department for nausea, vomiting, and dysphagia. He eventually sought care for a subarachnoid hemorrhage and then died. After it was determined that rabies virus had been transmitted to multiple recipients of organs from this donor, it was noted that the donor had had other symptoms consistent with encephalitis and had been bitten by a bat (*9*).

Although it was thought that this type of rabies virus transmission was extremely uncommon, another cluster of donor-derived rabies encephalitis cases was reported from Germany soon after the 2004 cases. Recognition of the second cluster demonstrates the potential ability to improve clinical recognition and diagnosis of transplant-transmitted encephalitis through increased awareness generated from knowledge of previous transmission events (*24*).

In these 2 clusters of solid organ transplant–transmitted rabies cases, infection was attributed to bat and canine virus variants, respectively. Within 6 weeks of undergoing transplantation, all but 1 recipient had rabies symptoms and died; the transplant recipient who survived was from the German cluster and had been previously vaccinated against rabies (*9*,*24*). These observations suggested a high rate of infectivity and incubation periods of ≈6 weeks in unvaccinated recipients of solid organs from donors with rabies.

In 2013, another case of transplant-transmitted rabies was identified in the United States (*25*). A raccoon rabies virus variant was identified in the organ donor and the infected recipient. Signs and symptoms of rabies developed in the organ recipient ≈17 months after the transplantation (Figure 2); 3 unvaccinated recipients of organs from the same donor remained asymptomatic. These findings contrast with those from previously reported clusters of transplant-transmitted rabies cases. Postexposure prophylaxis was initiated in the asymptomatic organ transplant recipients, and protective neutralizing antibody responses developed in all 3, suggesting that raccoon rabies infection can be prevented in organ transplant recipients if timely preventive measures are implemented (*25*).

## Lymphocytic Choriomeningitis Virus

LCMV (family *Arenaviridae*) is an enveloped virus with 2 negative-stranded RNA segments (*26*). The disease is an uncommon, primarily rodentborne infection that occurs among persons who have substantial contact with infected small rodents (*27*). Infection in immunocompetent persons is believed to be asymptomatic, and it is generally mild and self-limited in persons in whom clinical disease develops (*27*). Severe meningoencephalitis has been reported among immunocompromised patients (*28*).

**Figure 3:**
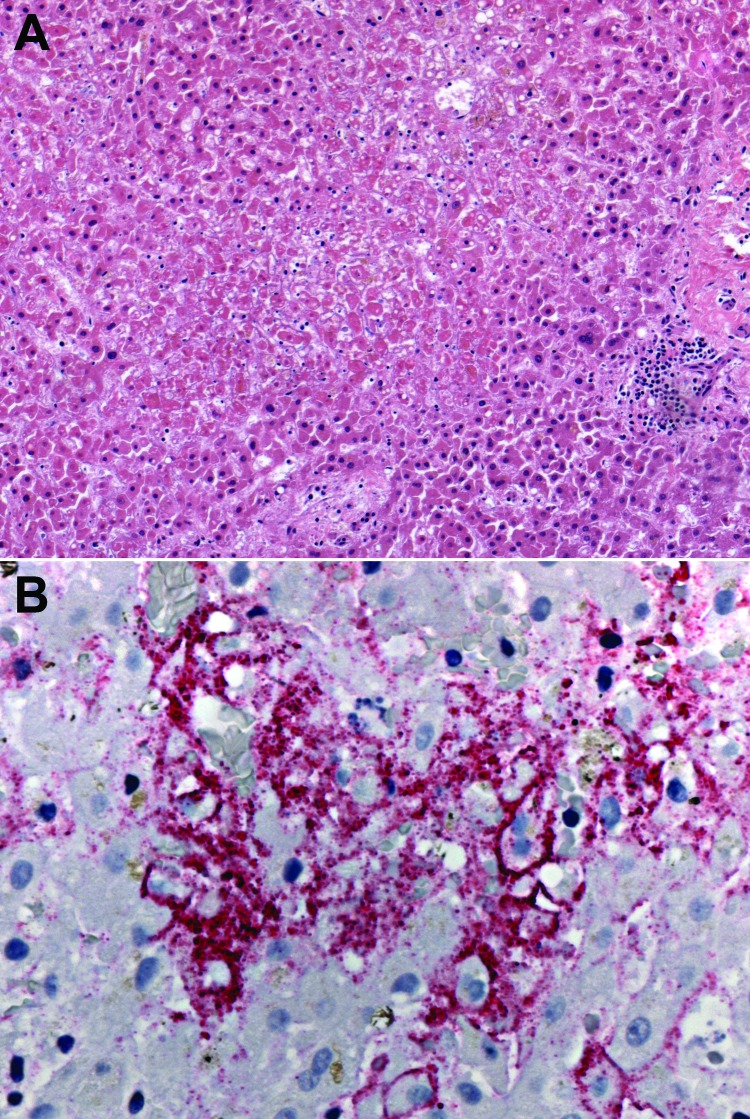
Photomicrographs showing histopathologic features and immunolocalization of lymphocytic choriomeningitis virus (LCMV) antigens in liver tissue from a liver transplant recipient with donor-derived LCMV infection. A) Massive hepatic necrosis, without prominent inflammation. Hematoxylin and eosin staining. Original magnification, ×50. B) LCMV antigens within hepatocytes and sinusoidal lining cells. Immunoalkaline phosphate staining, naphthol fast red substrate with light hematoxylin counterstain. Original magnification, ×158.

In 2003 and 2005, CDC investigated clusters of meningoencephalitis among solid organ transplant recipients in the United States (*29*). The 2003 cluster occurred among recipients of organs from a common donor from Wisconsin who had died from a subdural hematoma (*29*). There was no serologic evidence of LCMV infection in the donor and no evidence of exposure to rodents. LCMV was eventually detected in specimens from the donor by cell culture and electron microscopy evaluation, and the donor was identified as the source of the infections (*29*). The 2005 cluster of cases occurred among 4 recipients of organs from a common donor who died from an ischemic stroke (*30*). Pathologic investigation and immunohistochemical staining of specimens led to the diagnosis of LCMV infection in the transplant recipients (Figure 3), and epidemiologic investigation showed that the donor had a pet hamster (*10*). Although the donor had no evidence of active LCMV infection, the hamster was infected with a strain that was genetically similar to those that infected the transplant recipients.

In 2008, CDC investigated reports of hepatic insufficiency, multi-organ system failure, and death among 2 recipients of kidneys from a common organ donor (*31*). Although the recipients had no signs or symptoms of encephalitis, the donor had aseptic meningitis at death and was retrospectively found to have serum antibodies against LCMV. The donor was homeless and thus likely had multiple opportunities for exposure to rodents (*31*).

Another cluster of infections with a novel LCMV-related virus among 3 organ transplant recipients was reported in Australia; the patients died of encephalitis within 6 weeks of undergoing transplantation (*32*). In that cluster, the novel arenavirus was identified by the use of high-throughput sequencing with digital transcriptome subtraction (*32*). The donor, who died of a hemorrhagic stroke, had serologic evidence of recent LCMV infection and had recently traveled to rural southern Europe, where he may have been exposed to rodents (*32*).

These cases indicate that transplant-transmitted LCMV infection and encephalitis may be an uncommon, but quantifiable, infectious risk in organ transplantation. Immunosuppression in transplant recipients predisposes them to severe disease, even though infected donors may be asymptomatic.

## *Balamuthia mandrillaris* Amebae

*B. mandrillaris*, a species of small, free-living, aerobic amebae, has been reported as a cause of granulomatous amebic encephalitis (*33*). *B. mandrillaris*–associated skin lesions have been described, and untreated infection can progress to fatal encephalitis. Neurologic disease is characterized by the presence of single or multiple space-occupying intracranial lesions that cause a variety of focal and diffuse neurologic signs and symptoms (*33*). *B. mandrillaris*–associated encephalitis is almost always fatal, even with treatment (*34*). Diagnosis has traditionally required culture of the organism or identification of amebic trophozoites or cysts from biopsy samples of affected tissue; however, a real-time PCR for use with cerebrospinal fluid samples is available (*34*). Unlike other free-living amebae, which are typically found in fresh water, the natural reservoir for *Balamuthia* amebae is believed to be the soil. Clinical infection has been described among immunosuppressed patients, alcoholics, and otherwise debilitated persons (*33*,*34*).

**Figure 4:**
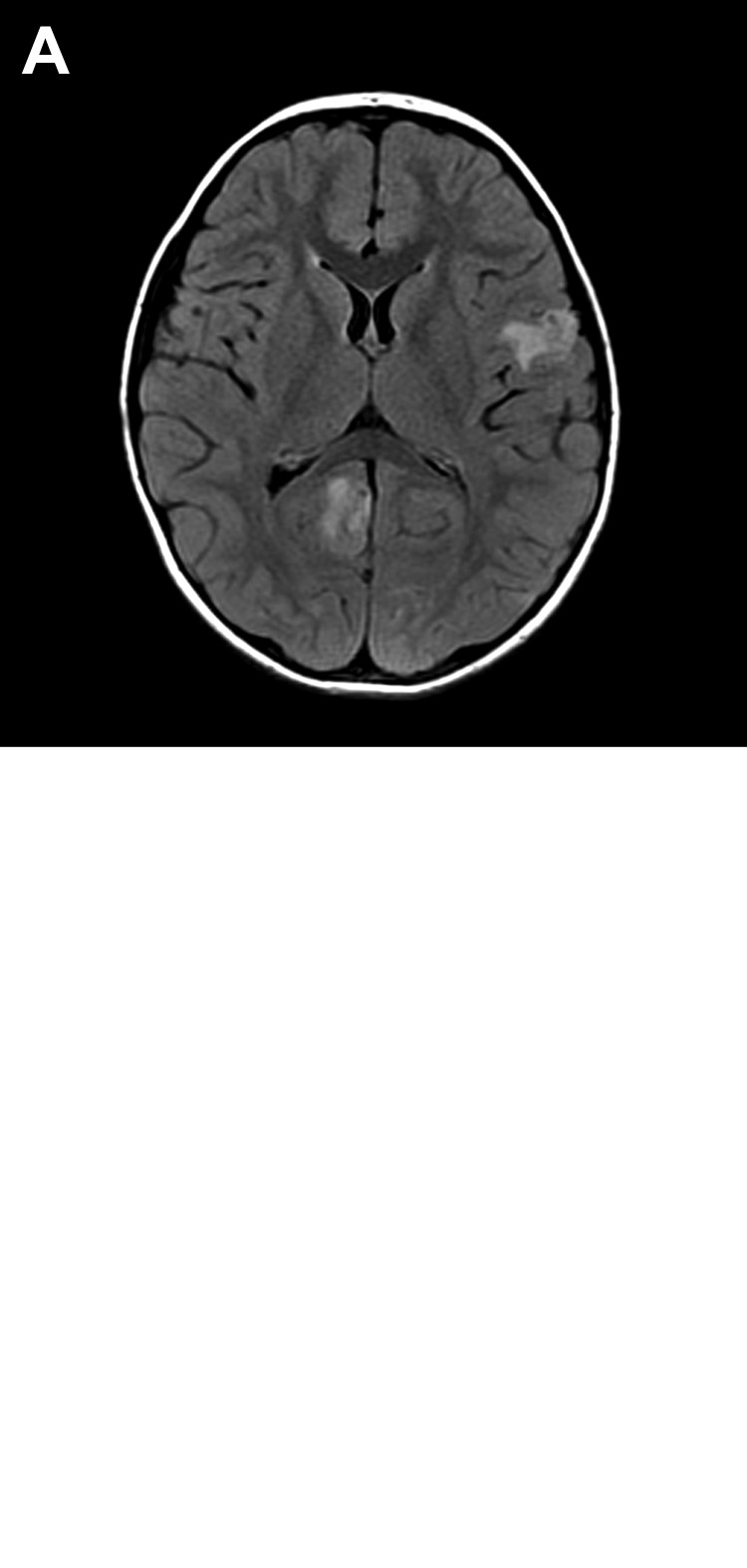
Brain images showing contrast-enhanced lesions in the right occipital and left parietal lobes of a 4-year-old boy with encephalitis caused by infection with *Balamuthia mandrillaris* amebae. A) T2-weighted fluid-attenuated inversion recovery (FLAIR) image. B) T1-weighted contrasted magnetic resonance image.

**Figure 5:**
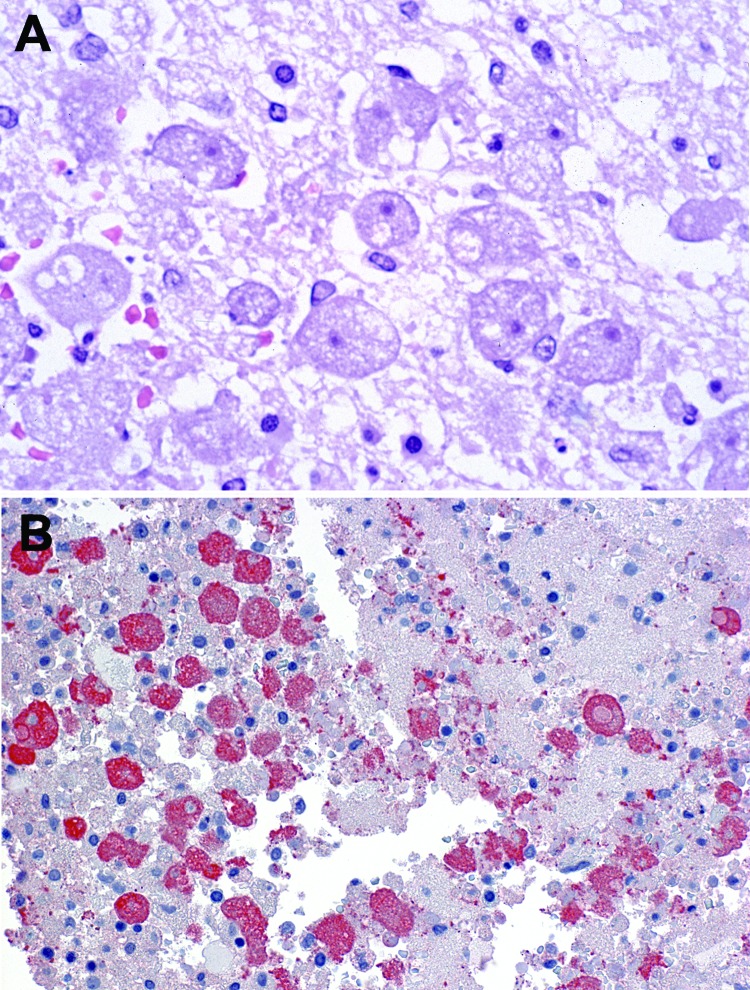
Photomicrographs showing histopathologic features and immunolocalization of *Balamuthia mandrillaris* antigens in central nervous system tissue from a donor with *B. mandrillaris* infection. A) Typical amebic trophozoites with prominent karyosomes in central nervous system. Hematoxylin and eosin staining. Original magnification, ×158. B) *B. mandrillaris* antigens in amebic trophozoites. Immunoalkaline phosphate staining, naphthol fast red substrate with light hematoxylin counterstain. Original magnification, ×100.

Infection with transplant-transmitted *B. mandrillaris* amebae was first identified by CDC in 2009 following reports by clinicians in Mississippi of encephalitis among 2 recipients of kidneys from a common donor (*11*). The 4-year-old organ donor reportedly had numerous exposures to soil and water before dying of seizures and subarachnoid hemorrhage. An initial diagnosis of immune-mediated encephalitis was made (Figure 4), but subsequent histopathologic evaluation and immunohistochemical testing showed that the child had *B. mandrillaris* infection (Figure 5) (*11*). A second cluster of infections with transplant-transmitted *B. mandrillaris* amebae was reported to CDC in 2010. In this cluster, encephalitis developed in 2 organ transplant recipients in Arizona; cerebral magnetic resonance imaging showed multiple ring-enhancing lesions (*35*). The organ donor was presumed to have died of a stroke. Both transplant recipients died, and a postmortem diagnosis was determined by immunohistochemistry and real-time PCR detection of *B. mandrillaris* amebae DNA in brain biopsy specimens (*35*).

## Discussion

The transplant-transmitted cases of encephalitis we described highlight several important diagnostic and clinical challenges related to the recognition and treatment of certain emerging infections (Table). The precise rate of donor-derived transmission of WNV, rabies virus, LCMV, and *B. mandrillaris* amebae that cause encephalitis among transplant recipients is not known, but such cases are rare and may not be immediately recognized by clinicians. Diagnosis is further complicated because of laboratory screening limitations for some of these pathogens. Moreover, few effective treatment options are available once patients exhibit signs or symptoms of infection. However, there is limited evidence that prophylaxis or treatment, even for asymptomatic transplant recipients, may be effective following exposure to these pathogens (*11*,*25*,*29*,*37*,*38*). Identification of possible infectious encephalitis among organ donors and establishment of proactive notification systems for transplant centers is crucial. Furthermore, surveillance systems for possible donor-derived infectious encephalitis could reduce illness and death among organ transplant recipients.

**Table:** Infectious agents associated with encephalitis reported among clusters of solid organ transplant recipients in the United States, 2002–2013*

Infectious agent	Classification	Natural transmission route	No. reported clusters	Clinical features of infection	Laboratory detection method	Treatment
WNV	Enveloped, positive, single-stranded RNA virus; *Flavivirus* family	Bites from infected mosquitoes (*Culex* spp.)	6	Febrile illness, meningitis, encephalitis, poliomyelitis-like limb paralysis	Detection of WNV-specific antibodies or WNV nucleic acid in serum or CSF samples	None; several experimental therapies under investigation
Rabies virus	Enveloped, negative, single-stranded RNA virus; *Rhabdoviridae* family, *Lyssavirus* genus	Exposure to secretions, typically saliva, of infected animals (in North America, most commonly bats, raccoons, and skunks)	2	Nonspecific prodrome followed by confusion, paresthesias, insomnia, agitation, paresis, spasm of swallowing muscles, coma, and death	Before death: PCR or virus isolation in saliva, PCR and fluorescent antibody testing of nuchal biopsy samples, antibody testing of serum, and PCR or antibody testing of CSF; after death: fluorescent antibody staining of brain tissue or frozen tissue from nuchal biopsy, and serologic diagnosis by neutralization tests in mice or cell culture	Supportive; treatment with induced coma and antiviral therapy, as reported (*36*); postexposure prophylaxis for asymptomatic recipients of organs from infected donors
LCMV	Enveloped, RNA virus; *Arenaviridae* family	Exposure to infected rodents, presumably to urine	3	Febrile illness in most symptomatic persons; aseptic meningitis, encephalitis	Cell culture, electron microscopy, immunohistochemistry, detection of LCMV antibodies, PCR or high-throughput sequencing	Supportive
*Balamuthia mandrillaris*	Free-living aerobic amebae	Ubiquitous in soil	2	Skin lesions; single or multiple space-occupying intracranial lesions; granulomatous amebic encephalitis characterized by hemiparesis, aphasia, seizures	Culture or identification of amebic trophozoites or cysts in biopsy sample of affected tissue; real-time PCR of CSF	Multidrug combinations, which may include pentamidine isethionate, 5-flucytosine, fluconazole, clarithromycin or azithromycin, sulfadiazine, miltefosine, thioridazine, or liposomal amphotericin B†

*CSF, cerebrospinal fluid; LCMV, lymphocytic choriomeningitis virus; WNV, West Nile virus. †http://www.cdc.gov/parasites/balamuthia/treatment.html.

Several difficulties are inherent in the identification and diagnosis of possible transplant-transmitted encephalitis. The hallmark clinical features of encephalitis (i.e., fever and altered mental status) are common in severely ill hospitalized patients, including persons who have undergone organ transplantation. Differentiating between encephalopathy caused by a severe underlying injury or illness and transplant-transmitted infection may be clinically challenging. It is therefore possible, that cases of encephalitis caused by transplant-transmitted pathogens may go unrecognized. Organs from a single donor are transplanted to recipients in multiple centers, frequently in widely separated geographic areas. Linking fever and encephalitis in transplant recipients to an infectious pathogen transmitted from a common donor may be relatively easy if all recipients are in the same hospital. Such a coincidence facilitated recognition of many of the clusters referenced here (*8*,*9*,*11*,*29*). However, if recipients are located in widely dispersed geographic areas, this linkage may not be recognized. Even when transplant-transmitted encephalitis is suspected, establishing a donor or recipient diagnosis may be challenging. Diagnosis of the infections that we described required specialized laboratory techniques, capacity, and training, all of which may not be available in most transplant centers. Furthermore, many laboratory techniques are not standardized. Even where testing is possible, transplant recipient samples may be unavailable or difficult to obtain, particularly if a brain biopsy specimen is required to establish a diagnosis. In some cases, donor specimens may not be available for definitive testing, thus leaving unanswered the possibility of a common etiologic agent.

Better recognition of encephalitis among organ donors and prompt notification of transplant centers regarding possible transmission are essential to improve clinical management of recipients, including prophylaxis or treatment. However, because of several logistical issues, it is currently challenging to recognize encephalitis in organ donors. Clinical, demographic, and social histories are often obtained from family members of deceased donors who may not have access to or recall historic details associated with the donor.

Further complicating organ safety in the United States is the regulatory oversight of solid organs. Although some policies are set by the Health Resources and Services Administration through the Organ Procurement and Transplantation Network, and infectious disease guidelines are available, screening of potential organ donors is under the purview of the individual organ procurement organizations, and variability exists in the testing that is performed for many agents (*39*). No organ procurement organization currently screens for all of the diseases discussed in this review. Given that the diseases are rare, laboratory screening of all donors is unlikely to be cost effective. However, introduction of a standardized risk assessment tool to gather medical, demographic, and social risk factors for infectious encephalitis from all organ donors may reduce the risk of transmitting infectious pathogens to transplant recipients. If a donor is perceived as a high risk for transmitting an infectious pathogen, transplant centers could be informed through a standardized proactive, notification system so that appropriate clinical management of transplant recipients could be considered.

The challenges encountered in investigating the clusters described here highlight the need for establishing surveillance systems for infectious illnesses among transplant recipients. Although infectious disease reporting systems already exist and potential cases of donor-derived infections are reviewed by the Organ Procurement and Transplantation Network Disease Transmission Advisory Committee, there is a need for a national system for rapid communication on disease clusters, particularly infectious encephalitis, involving organ and tissue recipients from a common donor. Given that prevention and treatment options are available, recognition of donor or recipient infection, even after transplantation, could improve clinical outcomes among recipients. In many of the detected transmission clusters, the implicated pathogen could not be identified in the donor, despite intensive examination. In others, the pathogen was identified in procured organs but could not be detected in the archived serum sample, which was readily available for testing. Rapid recognition of disease transmission in transplant recipients could facilitate intervention in recipients of a common donor, particularly if tissue, which may be transplanted in up to 100 recipients, has also been donated.

 Availability of appropriate samples to test is also a critical issue. The Retrovirus Epidemiology Donor Study Allogeneic Donor and Recipient (RADAR) repository, which was previously instituted in the blood transfusion community, could serve as an example of potentially effective surveillance linking organ donors and recipients (*40*). During 2000–2003, the RADAR repository contained pre- and posttransfusion specimens from 3,575 surgical patients. The specimens were linked to 13,201 blood donation samples and used to study the transmission of emerging infections (*40*). Such a repository, with voluntary participation, if linked with a surveillance system for transplant-transmitted encephalitis could obviate difficulties related to specimen availability and laboratory testing, facilitate diagnosis, better inform clinical management, and guide policy decisions related to organ donor guidelines. Cost and informed consent considerations may result in challenges to implementation of a specimen repository. All of the emerging infections described in this review were initially recognized by histopathologic evaluation and immunohistochemical testing of both autopsy and biopsy tissues. Increased rates of autopsy among organ donors could enable the identification of new and emerging infections in transplant recipients.

The investigation of infections resulting from organ transplant–transmitted pathogens requires rapid communication among transplant physicians, organ procurement organizations, and public health authorities. The presence of an unusual syndrome of signs and symptoms, including fever and change in mental status, particularly in the first few weeks following transplantation, should alert clinicians to the possibility of encephalitis caused by a transplant-transmitted infectious agent. Prompt notification to public health authorities can enable rapid investigation and discovery of clusters from a common donor. Until active surveillance can be implemented, timely communication and use of traditional and novel diagnostic testing can be crucial in identifying unusual and emerging infections caused by transplant-transmitted pathogens.
